# The relationship between circulating irisin, retinol binding protein-4, adiponectin and inflammatory mediators in patients with metabolic syndrome

**DOI:** 10.1590/2359-3997000000289

**Published:** 2017-09-04

**Authors:** Omur Tabak, Gonul Simsek, Fusun Erdenen, Volkan Sozer, Tuna Hasoglu, Remise Gelisgen, Esma Altunoglu, Cuneyt Muderrisoglu, Abdulhalim Senyigit, Hafize Uzun

**Affiliations:** 1 Internal Medicine Clinic lstanbul Kanuni Sultan Suleyman Education and Research Hospital Istanbul Turkey Internal Medicine Clinic, lstanbul Kanuni Sultan Suleyman Education and Research Hospital, Istanbul, Turkey; 2 Department of Physiology Istanbul University Cerrahpasa Medical Faculty Istanbul Turkey Department of Physiology, Istanbul University, Cerrahpasa Medical Faculty, Istanbul, Turkey; 3 Istanbul Education and Research Hospital Internal Medical Clinic Istanbul Turkey Istanbul Education and Research Hospital, Internal Medical Clinic, Istanbul, Turkey; 4 Department of Biochemistry Yildiz Technical University Istanbul Turkey Department of Biochemistry, Yildiz Technical University, Istanbul, Turkey; 5 Istanbul Medical Faculty Istanbul University Istanbul Turkey Istanbul Medical Faculty, Istanbul University, Istanbul, Turkey; 6 Department of Biochemistry Istanbul University Cerrahpasa Medical Faculty Istanbul Turkey Department of Biochemistry, Istanbul University, Cerrahpasa Medical Faculty, Istanbul, Turkey; 7 Medicine Hospital Internal Medical Clinic Istanbul Turkey Medicine Hospital, Internal Medical Clinic, Istanbul, Turkey

**Keywords:** Metabolic syndrome, high sensitivity C-reactive protein, pentraxin-3, interleukin-33, irisin, retinol binding protein-4, adiponectin

## Abstract

**Objective:**

We wanted to investigate whether there is a relationship between circulating irisin, retinol binding protein-4 (RBP-4), adiponectin and proinflammatory mediators implicated in the development of insulin resistance (IR) in metabolic syndrome (MetS).

**Subjects and methods:**

In 180 individuals, including controls and patients with MetS, we measured fasting plasma insulin, high sensitivity C-reactive protein (hsCRP), pentraxin-3 (PTX-3), interleukin-33 (IL-33), irisin, RBP-4, and adiponectin using ELISA kits.

**Results:**

While fasting plasma hsCRP, PTX-3, IL-33, irisin, RBP-4 concentrations were higher, adiponectin levels were lower in patients with MetS than in controls. A correlation analysis revealed that plasma irisin levels were positively associated with MetS components such as waist circumference and waist-hip ratio, low density lipoprotein (LDL) and markers of systemic inflammation such as PTX-3, hsCRP, uric acid, and RBP-4. Adiponectin levels were negatively associated with waist circumference, waist-hip ratio, PTX-3 and LDL.

**Conclusions:**

Although the precise mechanisms are still unclear, irisin, RBP-4, adiponectin and PTX-3 are hallmarks of the MetS, which is related to low-grade inflammation. It is conceivable that irisin and adiponectin might contribute to the development of MetS and may also represent novel MetS components. Future clinical studies are needed to confirm and extend these data.

## INTRODUCTION

The metabolic syndrome (MetS), which has an important role in cardiovascular morbidity and mortality, is generally characterized by obesity, insulin resistance (IR), hypertension, cardiovascular disease (CVD), non-alcoholic fatty liver disease and/or a proinflammatory state due to the accumulation of adipose tissue. Visceral obesity, which is central to MetS, leads to altered adipokines, IR, endothelial dysfunction and atherogenesis. When adipose tissue inflammation and dysfunction are established, adipokine secretion is significantly skewed toward a diabetogenic, proinflammatory and atherogenic pattern (1,2).

Irisin is a novel exercise-mediated myokine that regulates energy metabolism by increasing metabolic rate and mitochondrial content in both myocytes and adipocytes and plays an important role in metabolic diseases. Irisin acts as an exercise-induced insulin sensitizing hormone. The main effects of irisin are the browning of white adipose tissue and increased energy expenditure. Irisin expression and/or circulating levels have been associated with anthropometric and biochemical parameters, other hormones and adipokines, and obesity, IR, type 2 diabetes and MetS (3,4).

Vitamin A is an essential nutrient that is mainly stored in the liver. Retinol binding protein (RBP) is a plasma transporter that carries retinol from the liver to the periphery. Very little RBP originates from adipose tissue. RBP-4 is a novel adipokine and a negative acute phase reactant (5). The high concentration of RBP-4 plays a role in the progression of IR through immunity and inflammatory mechanisms in adipose and vascular tissues. RBP-4 seems to be a cardiometabolic marker in chronic inflammatory diseases including obesity, type 2 diabetes, MetS and CVD. These effects result from the direct activation of antigen presenting cells by RBP-4 (6).

Cytokines released by adipose tissue are involved in initiating and promoting a proinflammatory status, which contributes to IR. One of these cytokines, adiponectin, is suggested to sensitize the body to insulin and has a cardioprotective effect (2). Adiponectin is produced by adipocytes and has insulin sensitizing, anti-inflammatory, antioxidative and antiapoptopic properties. Adiponectin enhances insulin secretion by stimulating both the expression of the insulin gene and the exocytosis of insulin granules. Adiponectin also acts in the brain to increase energy expenditure and may thereby promote weight loss. Adiponectin is independently and negatively related to MetS, IR, type 2 diabetes, body weight, blood pressure and serum lipids (7-10).

Pentraxins are divided into the short and long pentraxin families. CRP is the prototype of the short pentraxin subfamily and pentraxin-3 (PTX-3) belongs to the long pentraxin family. PTX-3 is a multimeric acute phase inflammatory glycoprotein in the same family as CRP, a well-known cardiovascular biomarker (11). Its secretion is stimulated in endothelial cells, macrophages, myeloid cells, and dendritic cells by cytokines and endotoxins. After release, PTX-3 binds with C1q to initiate complement activation and facilitate pathogen recognition by macrophages. PTX-3 levels are increased in CVD. Although PTX-3 is an acute inflammatory protein in the same family as CRP, its levels may more directly reflect the inflammatory status of the vasculature (12,13).

Interleukin-33 (also known as IL-1) is a newly identified cytokine. Its receptor, soluble ST-2, has been shown to be protective in certain conditions such as obesity and atherosclerosis. IL-33 exerts protective metabolic effects by decreasing IR, leading to the accumulation of protective Th2 cells and cytokines and consequently reducing adipogenesis (14,15).

We aimed to investigate whether there is a relationship between circulating irisin, RBP-4, PTX-3, IL-33 and adiponectin along with the anthropomorphic and biochemical variables implicated in the development of IR in MetS.

## SUBJECTS AND METHODS

### Subjects

A total of 180 patients and control subjects between the ages of 30-65 were included in the study. MetS was diagnosed according to the Adult Treatment Panel III (ATPIII) criteria by the same physician (16). The components of MetS were waist circumference (WC) > 102 cm for males and > 88 cm for females, hypertension (HT) (systolic blood pressure (SBP > 130 mmHg, diastolic blood pressure (DBP) > 85 mmHg), treatment with antihypertensive drugs, high density lipoprotein- cholesterol (HDL-C) < 40 mg/dL in males and < 50 mg/dL in females, hypertriglyceridemia (TG) > 150 mg/dL, fasting blood glucose (FBG) > 100 mg/dL or the presence of type 2 diabetes mellitus (DM). MetS was defined as the presence of at least three components. Pregnant women, subjects with endocrine disorders, chronic cardiovascular, renal, hepatic, rheumatic diseases, smokers and subjects who were taking drugs which could affect our results were excluded. The control group consisted of 50 healthy people who were hospital staff. This group did not have diabetes, dyslipidemia or glucose intolerance as confirmed by an oral glucose tolerance test (OGTT). They neither had hepatic nor renal disease and were not taking drugs to affect carbohydrate metabolism. Pregnant women, patients with acute vascular or infectious illness or malignancy were also excluded. All subjects were of Turkish descent. The weight and height of each person were measured, and body mass index (BMI) was calculated according to the following formula: weight/height (m^2^). Waist circumference was measured with a flexible tape measurer at the level of the navel.

All participants were informed about the survey and freely signed and dated the consent form. The protocol was approved by the Ethics Committee of Istanbul Education and Research Hospital and was conducted in accordance with the Declaration of Helsinki (No: 2015/628).

### Laboratory analysis

#### Sample collection and preparation

Drugs were administered at least 24 h prior to blood collection. Clinical parameters, including routine biochemical parameters, were measured using standard protocols. Blood samples were collected in EDTA-containing tubes and anticoagulant-free tubes after an overnight fast. After immediate centrifugation (3000 g) for 10 min at 4 °C, plasma and serum were separated in Eppendorf tubes and frozen immediately at -80 °C until analysis.

The homeostasis model assessment (HOMA) was used to detect the degree of insulin resistance (IR) by measuring the levels of basal (fasting) glucose and insulin. HOMA-IR was calculated using the following formula: HOMA-IR = (fasting glucose [mg/dL] x fasting insulin [μU/mL]) / 405.

#### Measurement of plasma pentraxin-3 (PTX3) concentrations

Plasma PTX-3 levels were measured by a commercially available competitive enzyme-linked immunoassay kit (Hycult Biotech, Netherlands, Catno; HK347). The coefficients of intra- and inter-assay variation were 4.4% (n = 15) and 5.5% (n = 15), respectively.

#### Measurement of serum interleukin-33 (IL-33) concentrations

Serum Il-33 levels were measured by a commercially available competitive enzyme-linked immunoassay kit (eBiosience, Austria, BMS2048). The coefficients of intra- and inter-assay variation were 5.0% (n = 15) and 6.5% (n = 15), respectively.

#### Measurement of serum irisin concentrations

Serum irisin levels were measured by a commercially available competitive enzyme-linked immunoassay kit (EASTBIOPHARM, Hangzhou Eastbiopharm Co. Ltd. China). The coefficients of intra- and inter-assay variation were 6.7% (n = 15) and 7.5% (n = 15), respectively.

#### Measurement of serum retinol binding protein-4 (RBP-4)

Serum RBP-4 levels were measured by a commercially available competitive enzyme-linked immunoassay kit (EASTBIOPHARM, Hangzhou Eastbiopharm Co. Ltd. China). The coefficients of intra- and inter-assay variation were 6.4% (n = 15) and 7.2% (n = 15), respectively.

#### Measurement of serum adiponectin concentrations

Serum adiponectin levels were measured by a commercially available sandwich enzyme-linked immunoassay kit (DRG International, Inc., Marburg, Germany). The coefficients of intra- and inter-assay variation were 6.3% (n = 15) and 7.3% (n = 15), respectively.

Glucose, TC, TG, HDL-C and LDL-C concentrations were determined by enzymatic methods (Abbott Diagnostics, Abbott Park, IL, USA). The intra-assay and inter-assay coefﬁcients of variation were 2.6% and 3.0% for the assayed glucose, respectively, 2.6% and 3.0% for the total cholesterol assayed, respectively, 2.7% and 3.5% for the assayed TG, respectively, 3.5% and 4.1% for the assayed HDL-C, respectively, and 3.9% and 4.2% for the assayed LDL-C, respectively. Insulin concentrations were measured by an electrochemiluminescence immunoassay (ECLIA) method on a Roche-Hitachi E170. IR was calculated using the homeostasis model assessment formula (HOMA-IR, fasting insulin (mU/L) * glucose (mmol/dL/22.5). Analysis of CRP was performed by nephelometric means (IMAG-Bechman Coulter, Krefeld, Germany).

## Statistical analysis

Statistical analyses were performed using SPSS 20.0 for Windows (SPSS, Inc., Chicago, Illinois). The results are expressed as the mean ± standard deviation. An independent samples t-test was used to compare the mean values between the groups. Spearman’s rho test was used to determine the correlations with MetS risk factors. Pearson’s correlation was used for numerical data. In addition, a linear regression analysis was conducted to determine the parameters that significantly affect the MetS. Chi square test was performed to observe the quantitative similarity between genders. To assess the diagnostic accuracy, we performed receiver operating characteristic (ROC) curve analysis. ROC analysis was performed using MedCalc Statistical Software version 14.8.1 ((MedCalcSoftware bvba, Ostend, Belgium). The area under the ROC curve (AUC) was then estimated. *p* < 0.05 values were considered to be statistically significant.

## RESULTS

The demographic and biochemical values of MetS patients and controls are shown in Table 1. Age and sex matched groups revealed significant differences with regard to their demographic and biochemical values, as expected (Table 1). The comparison of irisin, retinol binding protein-4, adiponectin and inflammatory mediators of the groups according to HbA1c levels are shown in Table 2. Circulating irisin, RBP-4, adiponectin and inflammatory mediators of are shown in Table 3.

**Table 1:** Demographic and biochemical values of metabolic syndrome patients and controls

	Control group (n = 50)	Metabolic syndrome (n = 130)	P
Age	48.72 ± 5.98	50.29 ± 5.67	NS
Sex (F/M)	26/24	68/62	NS
Waist circumference (cm)	82.62 ± 9.13	102.87 ± 8.21	< 0.001
Hip circumference (cm)	96.82 ± 8.76	113.60 ± 9.19	< 0.001
BMI (kg/m^2^)	23.86 ± 2.40	33.54 ± 4.35	< 0.001
Systolic Blood Pressure (mmHg)	114.80 ± 8.06	137.08 ± 11.71	< 0.001
Diastolic Blood Pressure (mmHg)	78.40 ± 8.57	85.23 ± 8.06	< 0.001
Total protein (g/dL)	7.32 ± 0.40	7.46 ± 0.36	NS
Albumin (g/dL)	4.54 ± 0.29	4.42 ± 0.30	NS
Uric acid (mg/dL)	3.94 ± 1.00	5.35 ± 1.57	< 0.001
Total cholesterol (mg/dL)	162.50 ± 24.88	201.86 ± 46.22	< 0.001
HDL-C (mg/dL)	49.68 ± 9.52	46.18 ± 11	< 0.05
LDL-C (mg/dL)	97.84 ± 24.35	110.98 ± 37.49	< 0.01
Triglyceride (mg/dL)	94.00 ± 30.33	210.82 ± 112.08	< 0.001
Fibrinogen (mg/dL)	260.54 ± 54.07	330.68 ± 69.26	< 0.001
Glucose (mg/dL)	91.38 ± 9.29	177.15 ± 67.89	< 0.001
C-peptide (ng/mL)	1.73 ± 0.61	2.22 ± 1.14	< 0.01
HbA1_C_ (%)	5.51 ± 0.44	8.10 ± 1.73	< 0.001
Insulin (µU/mL)	9.51 ± 5.03	27.45 ± 30.79	< 0.001
HOMA-IR	2.16 ± 1.20	12.32 ± 16.82	< 0.001

**Table 2:** BMI: body mass index; LDL: low density lipoprotein; HDL: high density lipoprotein; NS: non-significant. . Comparison of irisin, retinol binding protein-4, adiponectin and inflammatory mediators of the groups according to HbA1c levels

	Group A (HbA1c < 6.5%) n = 68	Group B (HbA1c: 6.5-8%) n = 56	Group C (HbA1c > 8%) n = 56	P (A-B)	P (A-C)
hsCRP (mg/dL)	0.97 ± 1.37	2.97 ± 1.63	3.19 ± 1.72	**0.000**	**0.000**
PTX-3 (pg/mL)	256.89 ± 120.27	326.53 ± 107.10	317.83 ± 112.30	**0.001**	**0.005**
IL-33 (pg/mL)	5.18 ± 1.14	5.23 ± 0.82	5.37 ± 0.86	0.793	0.325
Irisin (ng/mL)	47.95 ± 19.05	62.61 ± 18.07	62.56 ± 17.23	**0.000**	**0.000**
RBP- 4 (mg/mL)	40.35 ± 12.28	54.49 ± 10.71	57.64 ± 15.51	**0.000**	**0.000**
Adiponectin (mg/mL)	6.13 ± 2.14	3.61 ± 1.27	3.46 ± 1.30	**0.000**	**0.000**

hsCRP: high sensitivity C-reactive protein; PTX-3: pentraxin-3; IL-33: interleukin-33; RBP- 4: retinol binding protein – 4.

There was no difference between Group B and C with regard to these biomarkers.

**Table 3:** Circulating irisin, retinol binding protein-4, adiponectin and inflammatory mediators of MetS and controls

	Control group n = 50	Metabolic syndrome n = 130	*P*
hsCRP (mg/dL)	0.35 ± 0.24	3.04 ± 1.67	< 0.001
PTX-3 (pg/mL)	235.29 ± 111.08	321.46 ± 110.63	< 0.001
IL-33 (pg/mL)	5.02 ± 1.12	5.35 ± 0.88	< 0.05
Irisin (ng/mL)	4.03 ± 1.29	6.35 ± 1.75	< 0.001
RBP-4 (ng/mL)	34.55 ± 6.63	56.13 ± 12.83	< 0.001
Adiponectin (µg/mL)	7.03 ± 1.71	3.56 ± 1.23	< 0.001

hsCRP: high sensitivity C-reactive protein; PTX-3: pentraxin-3; IL-33: interleukin-33; RBP- 4: retinol binding protein – 4.

Correlation analysis between biochemical variables of the whole group and the MetS group are shown in the Tables 4 and 5.

**Table 4:** Correlation analysis of groups

	Irisin		Adiponectin		RBP-4		PTX-3	
			
			r	*p*	r	*p*	r	*p*
SBP			-0.55	< 0.001	0.467	0.0001		
DBP								
BMI	0.36	< 0.001	0.34	< 0.001	0.479	0.0001	0.229	< 0.002
WC	0.49	< 0.001	0.54	< 0.001	0.474	< 0.0001	0.229	< 0.002
WHR	0.34	< 0.001	0.23	0.02				
hsCRP	0.30	< 0.001						
HbA1c	0.28	< 0.001	0.49	< 0.001	0.487	< 0.0001	0.221	0.003
Glucose			0.47	< 0.001	0.51	< 0.0001		
Insulin			0.24	< 0.001				
HOMA-IR			0.26	< 0.001	0.258	< 0.0001		
Cholesterol			0.40	< 0.001	0.656	< 0.0001	0.508	< 0.0001
LDL-C	0.01	0.88	0.29	< 0.001	0.596	< 0.0001	0.555	< 0.0001
Uric acid	0.19	0.01			0.559	< 0.0001	0.416	< 0.0001
Fibrinogen			0.36	< 0.001	0.349	< 0.0001		
PTX-3	0.16	0.16	0.34	< 0.001	0.512	< 0.0001		
RBP-4	0.25	0.001						
Irisin			0.36	< 0.001	0.253	< 0.001		

**Table 5:** Correlation analysis in MetS patients

	r	*p*
Uric acid-Cholesterol	0.541	0.0001
Uric acid-LDL-C	0.606	0.0001
Uric acid-hsCRP	0.454	0.0001
Uric acid-PTX-3	0.414	0.0001
Uric acid-PBB-4	0.485	0.0001
Uric acid-Adiponectin	-0.246	0.0001
LDL-hsCRP	0.652	0.0001
LDL-PTX-3	0.638	0.0001
LDL-RBP	0.694	0.0001
LDL-Adiponectin	-0.323	0.0001
hsCRP-PTX-3	0.472	0.0001
hsCRP-RBP-4	0.521	0.0001
hsCRP-Adiponectin	-0.219	0.012
PTX-3-RBP4	0.487	0.0001
PTX-3-Adiponectin	-0.373	0.0001
RBP-4-Adiponectin	-0.215	0.014
Irisin-WC	0.246	0.005
Irisin-WHR	0.468	0.0001
Adiponectin-SBP	-0.208	0.017

Regression analysis for irisin revealed that only WC had a significant effect on MetS (p = 0.02). WHR, BMI, HOMA-IR, uric acid, LDL-C, hsCRP, PTX-3, IL-33, RBP-4, and adiponectin levels did not correlate with irisin. Adiponectin levels did not correlate with these parameters in MetS. Correlation analyses of the new biomarkers are shown in Table 6.

**Table 6:** Correlation analysis of the new parameters with each other

			Irisin	PTX-3	RBP-4	Adiponectin	hsCRP
MetS	RBP-4	r		0.487		-0.215	0.521
		p		0.0001		0.014	< 0.0001
	PTX-3	r				-0.373	0.472
		p				< 0.0001	< 0.0001
	Adiponectin	r					-0.219
		p					0.012
Whole	Irisin	r			0.253	-0.363	0.299
		p			0.001	0.0001	0.0001
	RBP-4	r		0.512		-0.567	0.702
		p		0.0001		0.0001	0.0001
	PTX-3	r	0.157			-0.339	0.502
		p	0.035			0.0001	0.0001
	Adiponectin	r	-0.363	-0.339			-0.566
		p	0.0001	0.0001			0.0001
	IL-33	r		0.153			
		p		0.041			

**Figure 1:**
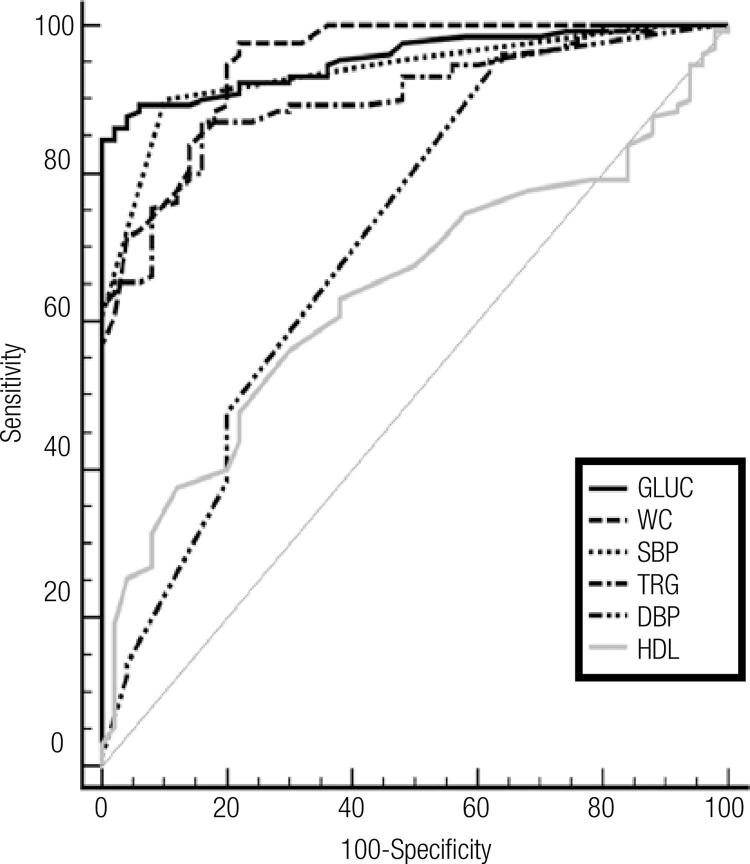
ROC curves of MetS parameters.

**Figure 2:**
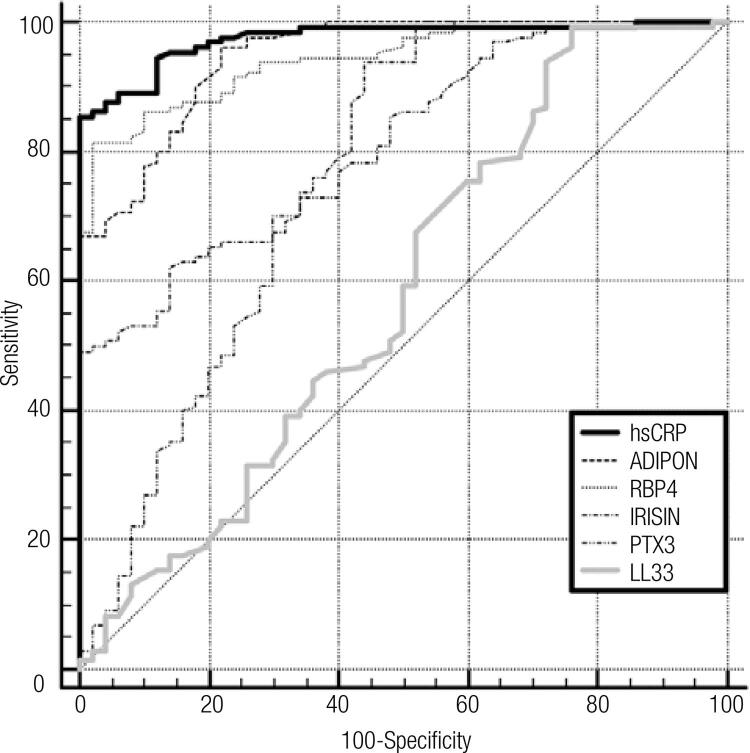
ROC curves of the new biomarkers.

A comparison of the ROC curves with sensitivity, speciﬁcity, AUC, cut-off and asymptotic signiﬁcance of systolic BP, DBP, WC, glucose, TG, HDL-C, hsCRP, irisin, RBP-4, PTX-3, adiponectin and IL-33 levels in the whole group are shown in Table 7 and in ROC curves in Figures 1 and 2.

**Table 7:** Sensitivity, specificity, cut-off, AUC and asymptotic significance of parameters

	Sensitivity (%)	Specificity (%)	Cut-off	AUC	SE	95% CI	Asymptotic sig.
Systolic BP	90.00	90.00	> 120	0.938	0.016	0.892-0.968	< 0.0001
BMI	96.15	94.00	> 27.18	0.980	0.010	0.947-0.995	< 0.0001
HbA1c	87.69	98.00	> 6.2	0.958	0.013	0.918-0.982	< 0.0001
HOMA-IR	93.08	80.00	> 2.68	0.927	0.019	0.878-0.960	< 0.0001
hsCRP	85.38	100.00	> 0.93	0.975	0.009	0.940-0.992	< 0.0001
RBP-4	81.54	98.00	> 44.56	0.945	0.015	0.901-0.973	< 0.0001

## DISCUSSION

Adipose tissue is an important source of adipokines that have proinflammatory effects and may be the link between obesity, CVD, diabetes and MetS (1,2). In our study, there was a statistically significant difference between the MetS group and the control group with respect to WC, WHR, BMI, blood pressure, lipid parameters, uric acid, fibrinogen, glucose, insulin, C-peptide and HbA1c values. Some of these variables are components of MetS, so this is not surprising. More importantly, there were significant differences with regard to irisin, RBP-4, IL-33, PTX-3 and hsCRP levels in favor of the MetS group; the opposite finding was observed for adiponectin.

We found that circulating irisin levels were positively associated with MetS components including BMI, WC and WHR, while in some studies, there were conflicting results regarding the relation between irisin levels with metabolic parameters. We also observed that women had lower levels of plasma irisin levels compared to men. In the control group, although women had higher levels, this was not statistically significant. We do not know the mechanism underlying the difference between the two genders. We suggest that this may be due to increased muscle mass, hormonal effects, the duration of obesity or drugs. hsCRP, IL-33, PTX-3, RBP-4 and fibrinogen levels did not change between males and females in the MetS group. Among other variables, only HDL-C levels were different between the two genders, as expected. Rizk and cols. (17) found that irisin levels were increased and positively correlated with BMI, serum triglycerides, HOMA-IR and liver enzymes in patients with MetS. Some authors showed a positive association between irisin levels and plasma glucose and TG levels in type 2 diabetic patients (18,19). Contradictory to these studies, Assyov and cols. (20) found a negative relationship with fasting glucose levels, although he observed a positive association of irisin with BMI. Wang and Li (21) reported decreased irisin levels in patients with type 2 DM compared with nondiabetics and suggested that irisin was not associated with beta cell function (21). We did not observe a positive relationship between irisin with plasma glucose, insulin and cholesterol levels. In a cohort of sedentary women, Duran and cols. (22) observed a negative correlation between irisin and BMI, postprandial glucose, LDL-C and TG levels, but a positive relationship with HDL-C. We suggest that irisin might be an early marker of MetS which only affected WC and WHR, but did not influence blood pressure and biochemical parameters.

IL-6 is secreted from adipose tissue in obesity and circulating levels of CRP are increased through adipocyte-derived IL-6 (23). We showed that hsCRP levels were positively correlated with PTX-3 and RBP-4, which are molecules involved in inflammatory conditions; hsCRP was negatively correlated with adiponectin. Inflammation in the vasculature might be an important pathogenic link between CVD and MetS. In our study, we found significantly higher levels of PTX-3 in MetS patients than in control subjects. There was a positive association between PTX-3 and irisin, RBP-4 levels and MetS components; a negative relationship was found between PTX-3 and adiponectin levels. A normal PTX-3 concentration was found to be approximately 2 ng/mL; men had significantly lower values than women (12,13). There was no difference between genders regarding PTX-3 levels in our study. We found an association of PTX-3 with risk factors such as obesity, uric acid, LDL-C, hsCRP, RBP-4 and adiponectin. Inoue and cols. (12) showed that the PTX-3 level was increased in the oldest age group and was also inversely correlated with triglyceride levels and BMI. It has also been found to be independent of other established coronary risk factors (13). Kardas and cols. (24) reported that PTX-3 levels were higher in obese children and adolescents with MetS and CVD, and that these levels were positively correlated with TG and negatively correlated with HDL-C levels (24,25). It was suggested that PTX3 levels may increase in order to confer protection against cardiac tissue damage. PTX3 binds to activated platelets and reduces inflammation in the cardiovascular bed (12), and might be a novel marker for subclinical atherosclerosis (25). These findings suggested that both PTX-3 and RBP-4 may be used to predict inflammatory status in MetS instead of hsCR, which is a well- known acute phase inflammatory marker. In accordance with some studies, we found that RBP-4 levels were higher in obese subjects than controls. We observed that RBP-4 was positively correlated with uric acid, LDL-C, hsCRP, and PTX-3, and negatively related to adiponectin. The concentration of RBP-4 was found to be elevated in obesity and type 2 DM, MetS and CVD (5). Many authors found that RBP-4 levels were associated with MetS parameters and HOMA-IR, the duration of diabetes and carotid atherosclerosis as determined by CIMT (26-29). There are conflicting results about the association of RBP-4 and CRP levels, probably due to methodological differences (30,31). Jialal and cols. suggested that serum irisin and RBP-4 levels would be independent predictors of CVD in diabetes (30). We think that RBP-4 is significantly associated with nearly all components of MetS and inflammation. It should be kept in mind that circulating RBP concentrations depend on vitamin A status; therefore, the serum retinol concentration may be a confounder (5). We did not measure vitamin A levels.

As expected, we found lower levels of adiponectin in MetS patients than controls. Adiponectin levels were negatively associated with SBP, WC, WHR, PTX-3, RBP-4, hsCRP, uric acid and LDL-C. Bidulescu and cols. (32) observed higher levels of adiponectin in African American women compared to men. Chiara and cols. (33) showed that subjects with low adiponectin levels had a higher prevalence of obesity, MetS, DM and CVD.

In our research, we found IL-33 levels were associated with HbA1c and insulin in the control group, but there no association of this marker with other variables. Interestingly, there was no association between IL-33 and adiponectin. IL-33 might be either proinflammatory or anti-inflammatory depending on the disease and the model. However, IL-33 was shown to have various protective effects in CVD, obesity and diabetes. Reduced levels may increase the risk of developing insulin resistance (14,15).

A strength of our study is that we evaluated multiple biomarkers involved in MetS and searched for the relationship between each of them with anthropomorphic and biochemical variables. However, our study has some limitations. First, our sample size is relatively small. Second, dietary habits, physical activity and the exercise level of the subjects were not documented. Third, we did not investigate cardiovascular comorbidities and drugs which could affect our results. Last, we did not measure vitamin A levels which might affect the RBP-4 level. Due to the cross-sectional design of our study, we cannot make any suggestions about the association between the laboratory and clinical parameters of the subjects. Prospective trials are needed to observe this relationship. We think that obesity induced cytokines initiate and promote a proinflammatory status leading to clinical consequences such as IR, DM, HT and atherosclerosis. Discrepancies in our results from previous studies may be due to different study populations or differences in their diet and exercise.

MetS is associated with impaired glucose homeostasis and low-grade chronic inflammation, as well as myokines and adipokines that interact through complex networks. Although the precise mechanisms are still unclear, elevated PTX-3, RBP-4, IL-33, irisin and decreased adiponectin levels increase the risk of obesity-related metabolic disorders. The inflammatory condition associated with overweight plays an important role in the components of the MetS and largely contributes to the related pathological outcomes. Our findings suggested that irisin might be an early marker of MetS that emerges before anthropomorphic, biochemical and clinical parameters. We also suggest that both PTX-3 and RBP-4 may be used to predict the inflammatory status in MetS instead of hsCR, which is a well-known acute phase inflammatory marker. The contradictory results between this study and others may be linked to the different stages of the MetS. Subjects should be evaluated prospectively with anthropomorphic, biochemical and clinical aspects at regular intervals. Future clinical studies are needed to confirm and extend these data.
